# Value of Ultrasonographic Optic Nerve Sheath Diameter in Assessing Increased Intracranial Pressure in Patients With Moderate to Severe Traumatic Brain Injury

**DOI:** 10.1186/2197-425X-3-S1-A488

**Published:** 2015-10-01

**Authors:** A Abdalla, BN Beshey, AM Abougabal, KM Elzahaby

**Affiliations:** Alexandria University, Faculty of Medicine, Critical Care, Alexandria, Egypt; Alexandria University, Faculty of Medicine, Radiodiagnosis, Alexandria, Egypt; Police Force Hospital, Intensive Care, Alexandria, Egypt

## Introduction

Among the factors associated with long-term outcome after TBI, intracranial pressure (ICP) confirms its relevance. of the non-invasive methods to assess raised ICP, ultrasound measures of optic nerve diameter seems especially promising and can be used in selected situations.

## Objectives

To determine the value of optic nerve sheath diameter measurement using ultrasonography in assessing increased intracranial pressure and outcome of moderate to severe traumatic brain injury patients.

## Methods

We enrolled 40 patients admitted over a period of 6 months with moderate to severe traumatic brain injury to the Department of Critical Care Medicine at the Alexandria Main University Hospital. ONSD measurements were performed by means of transorbital sonography daily for 7 days. Marshall and Rotterdam head CT neuroimaging scales were recorded on admission, 48 hours and 5 to 7 days later. Glasgow Outcome Scale (GOS) was assessed six months after discharge for survivors.

## Results

The mean ONSD upon admission (baseline value) was 6.29 ± 0.78 mm, increasing non-significantly on day 2 to 6.53 ± 0.53 mm (p = 0.546). However, on days 3, 4, and 5 the mean ONSD significantly increased to 6.63 ± 0.57 mm (p = 0.030), 6.72 ± 0.65 mm (p = 0.001), and 6.70 ± 0.71 mm (p = 0.003) respectively compared to the baseline value. a non-significant increase followed in days 6 and 7 to means of 6.59 ± 0.73 mm (p = 0.249), and 6.38 ± 0.72 mm (p = 1.000) respectively compared to the baseline value. the serial comparison of each mean ONSD to either the preceding or following daily mean was significant (p < 0.001). We couldn't demonstrate any correlation between ONSD and either of the neuroimaging scales used to evaluate intracranial hypertension; namely, Marshall Scale and Rotterdam Scale. ONSD could not predict mortality in our TBI patients.

## Conclusions

In the early posttraumatic period, transorbital ultrasound scans to measure ONSD may be useful for detecting high ICP after moderate severe TBI by providing additional non-invasive means of assessing ICP. However it doesnot predict mortality in the severe TBI patients.

**Figure 1:**
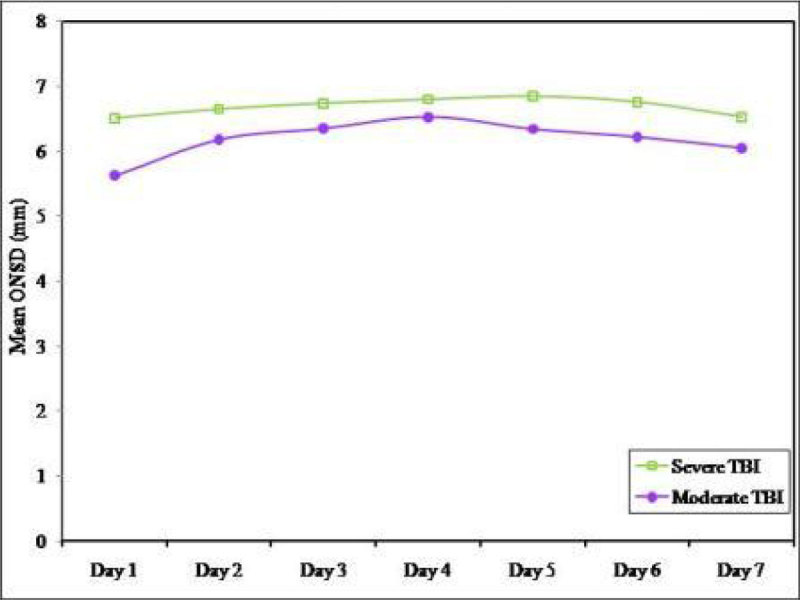
***Comparison of the mean ONSD of the studied cases over the period of seven days.***

**Figure 2:**
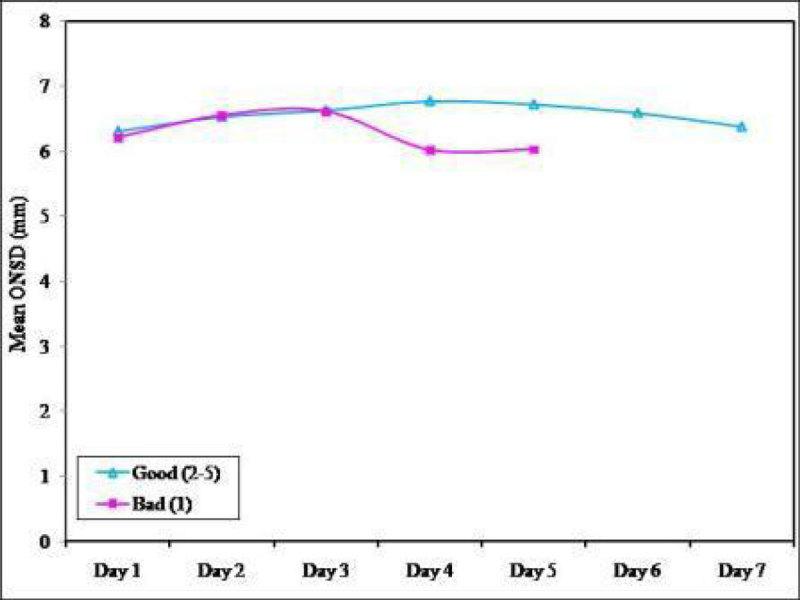
***Mean ONSD comparison of the studied cases over the period of seven days.***

**Figure 3:**
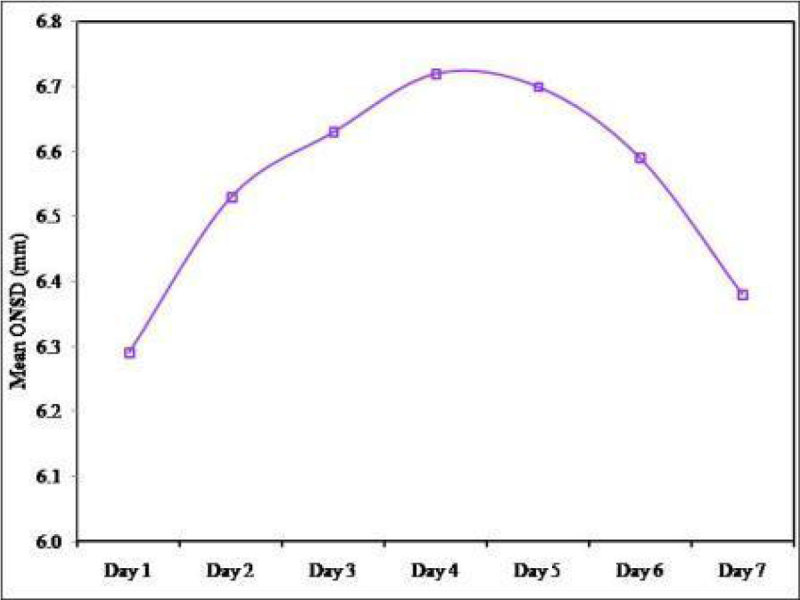
***Mean ONSD of the studied cases over the period of 7 days.***
